# The Central Role of Left Atrium in Heart Failure

**DOI:** 10.3389/fcvm.2021.704762

**Published:** 2021-08-13

**Authors:** Myriam Carpenito, Diego Fanti, Simona Mega, Giovanni Benfari, Maria Caterina Bono, Andrea Rossi, Flavio Luciano Ribichini, Francesco Grigioni

**Affiliations:** ^1^Unit of Cardiac Sciences, Department of Medicine, Campus Bio-Medico University of Rome, Rome, Italy; ^2^Section of Cardiology, Department of Medicine, University of Verona, Verona, Italy

**Keywords:** left atrium, heart failure, speckle-tracking echocardiography, left atrial strain, cardiovascular disease, left atrial function

## Abstract

In past cardiovascular medicine, the attention to the left ventricle-identified as the only indicator and determinant of healthy or unhealthy cardiac conditions- has systematically hidden the role of the left atrium (LA). The recent advances in cardiovascular imaging have provided a better understanding of LA anatomy, physiology, and pathology, making us realize that this functional structure is far from being an innocent spectator. We now know that the LA's mechanical and neuro-hormonal properties play a relevant part in several cardiovascular diseases, including atrial fibrillation, ischemic heart disease, valvular heart disease, and heart failure. The present review aims to describe the role of LA in the specific setting of heart failure. We provide currently available information on LA structure and function and summarize its role as a determinant of symptoms, prognosis, and potential therapeutic target in heart failure patients.

## Introduction

Heart failure (HF) with preserved ejection fraction (HFpEF) and reduced left ventricular (LV) ejection fraction (HFrEF) is associated with several structural and functional changes in the left atrium (LA). Until recently, the role of the LA in the development of HF was unclear. Traditionally, it was thought that this chamber modulated LV filling and cardiac output. New non-invasive imaging modalities have improved our understanding of the function and clinical impact of the LA (1, 2). Furthermore, the LA plays endocrine and regulatory roles closely related to its mechanical function (3), making it a potential treatment target and a predictor of cardiovascular events in a broad range of patient populations.

## Assessment of LA Size and Function

**Figure 1:**
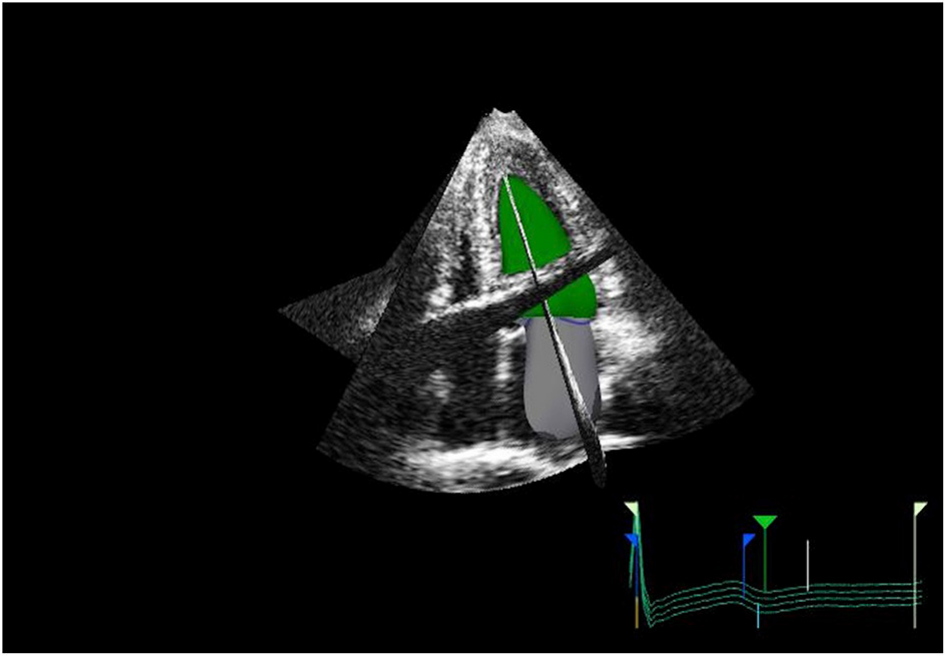
3D echo reconstruction of the left atrium (LA) and left ventricle (LV). LA is shown at its end-diastolic phase in order to appreciate left maximal atrial volume.

Measurement of LA size is a crucial element of a multiparametric assessment of patients with HF. LA size is measured with M-mode and two-dimensional transthoracic echocardiography (2DE) by evaluating the anteroposterior diameter (4). However, this has proven inaccurate, as the LA does not dilate uniformly. The maximal left atrial volume indexed to the body surface area (LAVi) is the method of choice as it is considered the most accurate. In fact, it is strongly associated with cardiac outcomes (5) and enables risk stratification. The predictive power of LAVi has been enhanced by the advent of three-dimensional echocardiography (3DE) (6, 7), which allows a more precise evaluation of the left atrial volume (LAV) without geometric assumptions and foreshortening (8) (Figure 1). Values from 3DE better correlate with the volume obtained with

cardiac magnetic resonance imaging (CMR) or cardiac computed tomography (CCT) (9). However, since CMR provides an adequate definition of the inner wall of the endocardium, and is able to detect pathological characteristics of the myocardial tissues, it is considered the gold standard in LAV measurement (10, 11).

**Figure 2:**
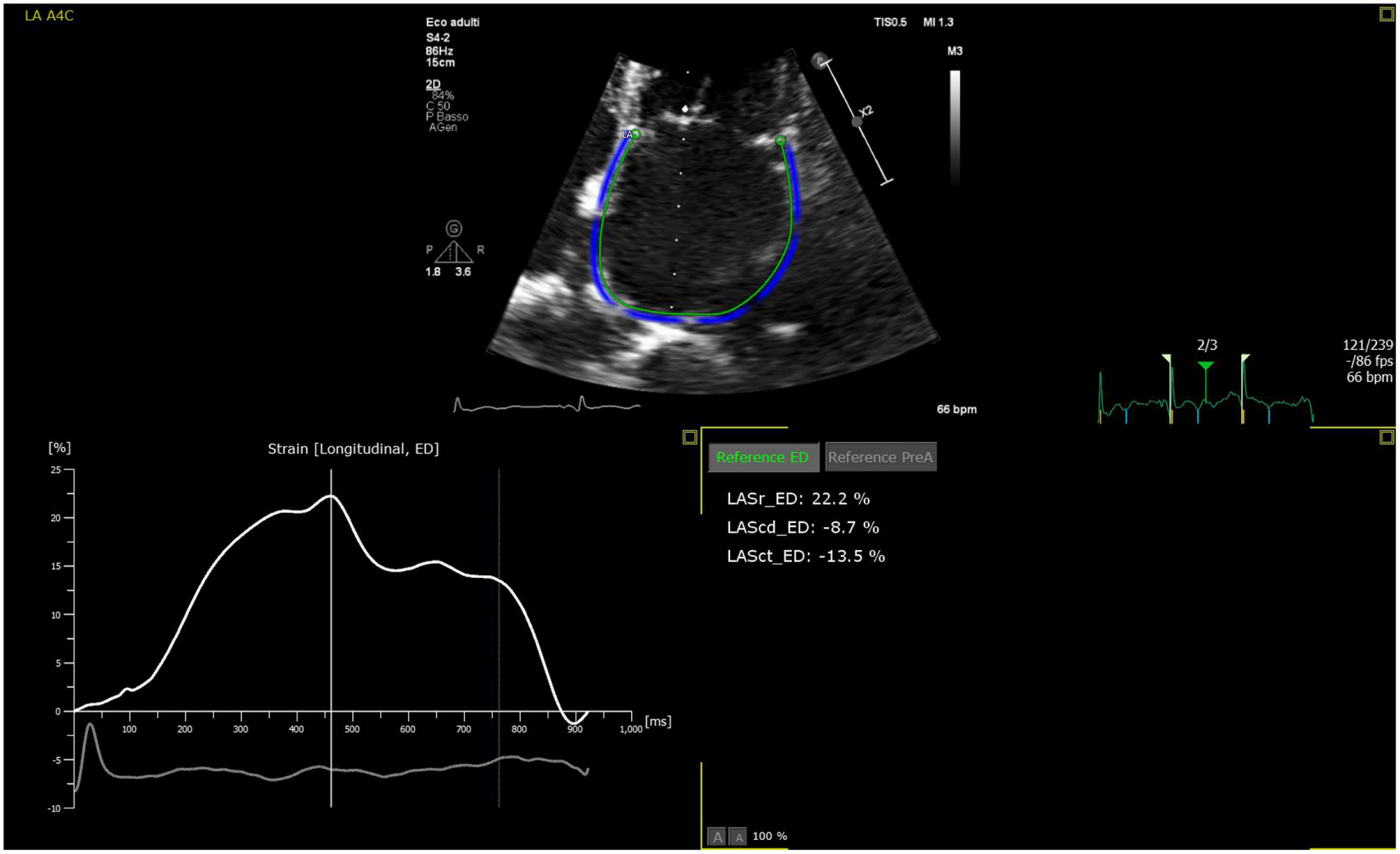
LA strain curve with R-R gating method in apical four-chamber view with zoom-in focus. The patient is an 83-year-old female with chronic heart failure. The patient is in sinus rhythm with reservoir function reduced. Zoomed image is used to increase the frame rate to enhance strain analysis accuracy.

The LA is a dynamic structure, and its mechanical function consists of three phases. It acts as a reservoir of oxygenated blood from the pulmonary veins, and its function depends on both the LV filling pressure and LV end-systolic volume; thus, any LV dysfunction will inevitably impact the atrial reservoir function (12). In early diastole, the LA acts as a conduit between the pulmonary veins and the left ventricle. The compliance of the atrial and ventricular chambers influences the conduit function of the LA, which is mutually related to the reservoir function (13). Usually, this phase makes a minor contribution to ventricular stroke volume but predominates in advanced stages of diastolic dysfunction, when the reservoir function upon atrial contraction is impaired (14, 15). Finally, the atrial booster pump function reflects atrial contractile function. It is dependent on intrinsic LA contractility, the degree of venous return, and LV end-diastolic pressures (16). Growing evidence suggests that the assessment of LA function provides more prognostic information than LA size in HF patients (17). The LA function can be evaluated through 3DE volumetric analysis by measuring all volumes from a single volume trace. Data relative to emptying volumes and fractions can be obtained by assessing the maximum, minimum, and pre-atrial contraction (immediately before) volumes. The transmitral spectral Doppler, pulmonary venous, and left atrial appendix flows also reflect phasic function but are currently rarely used (18). Alternatively, the phasic LA function can be derived from either tissue Doppler imaging (TDI) or 2D speckle-tracking echocardiography (2DSTE) (19, 20). Among these, 2DSTE is the most accurate method due to its ability to analyze myocardial deformation without angle dependency using frame-by-frame tracking of the speckles pattern generated by the interactions between ultrasound and myocardial tissue. The measurement of LA strain depends on whether the P wave (P-LASr) or the QRS (left atrial strain during reservoir phase, QRS-LASr) complex is used as the zero references. However, the recent European Association of Cardiovascular Imaging/American Society of Echocardiography recommends the use of QRS onset as the preferred method (21) mainly due to the impossibility of applying the P wave method to all patients, especially those with atrial fibrillation (AF) (22). The reservoir function is determined by the positive peak of atrial longitudinal strain (PALS), which indicates the LA's maximum elongation during LV systole (23). Hence, PALS also reflects the longitudinal contraction of the LV due to the interdependence between the LA and LV chambers (24). At the end of LA diastasis, there is a progressive shortening of the LA until the first negative peak of atrial contraction strain (PACS) or late diastolic strain. This event reflects the LA booster pump function (Figure 2).

## LA Remodeling and Mechanical Failure

LA dilatation is a compensatory mechanism required to maintain an average stroke volume, at least in the early stages of LV diastolic dysfunction (LVDD). LA dilatation reflects the chronicity and the severity of longstanding elevated LA pressure and LV high filling pressure (25, 26). It enhances conduit filling and initial improvement of its contractile function *via* the Frank-Starling effect. Modification of diastolic properties allows the LV to operate at a higher filling pressure during diastole; therefore, the relative contribution of the LA booster pump to LV filling increases until the LA preload reserve limits are reached. In this phase, LA behaves mainly like a conduit, and mechanical failure occurs (27). When the LA becomes dysfunctional, it loses its buffering effect, which leads to pulmonary congestion (28). Chronic exposure to elevated pressure leads to structural alterations of the LA (29), followed by myocyte hypertrophy, necrosis, apoptosis, and fibrosis. These events, along with altered ionic channel expression (30), contribute to electrical remodeling and the development of AF. LA dilatation is usually associated with annular dilatation and subsequent development of functional mitral regurgitation (MR), which even in the low range, contributes to the impairment of pulmonary hemodynamics (31) and the development of HF symptoms (32–34). LAV may also increase in some conditions characterized by normal diastolic function, such as in those with bradycardia, high-output states, atrial arrhythmias, and significant mitral valve disease, as well as trained athletes (35). The use of LA strain as a functional adaptive marker may provide valuable information on LA stiffness and indirectly estimate the LV end-diastolic pressure. It may identify atrial impairment at an early stage before dilatation occurs. Nevertheless, PALS needs to be employed with caution to identify isolated LA dysfunction (36) due to a close relationship between PALS and downward displacement of the LV base toward the apex and LV global longitudinal strain (GLS) (37).

## Pathophysiology of LA Mechanics in HF

### HFpEF

HFpEF is the most prevalent type of HF in the outpatient setting, accounting for more than half of all hospitalizations with decompensated HF (38). It is associated with elevated morbidity and high cardiovascular and non-cardiovascular mortality (39). Additionally, in contrast to HFrEF, there are no definitive therapies that improve outcomes in HFpEF (40). For the diagnosis of HFpEF, signs or symptoms of HF must be present. These include preserved ejection fraction (EF), elevated biohumoral markers with concomitant structural changes in the myocardium, and/or increased filling pressure (40). The LA is responsible for ~30% of physiological stroke volume, and functional impairment is a significant contributor to HF (41). However, it is still unclear when the transition from LVDD to HFpEF occurs and whether an alteration in atrial function contributes to this step. Diagnosis can be challenging because not all patients with LVDD present with HFpEF. Guidelines recommend a multiparametric stepwise diagnostic process (42). Echocardiography plays a central role in the diagnosis of HFpEF. Some authors suggest that E/e' and tricuspidal regurgitation velocity during exercise test may detect HFpEF with increased sensitivity (43). LA dilatation, which indicates chronically elevated LA pressure (44), is a predictor of hospitalization and mortality in HFpEF, particularly when associated with increased pulmonary pressure (45). Recent studies have proposed that the minimum LAV might better reflect LV end-diastolic pressure (46), particularly in patients without valvular disease or AF. A recent study compared atrial function in HFpEF and HFrEF patients with similar, invasively measured mean LA pressures (47). Patients with HFrEF presented larger LAVs, while those with HFpEF had higher pressure pulsatility, with more significant wall stress variation. These results may explain the origin of AF in patients with HFpEF, where it is more commonly observed, despite smaller LAVs. Chronically elevated LV filling pressures cause eccentric hypertrophy of the LA with consequent atrial endocrine failure (48, 49). LA dysfunction, evaluated by measurement of the LA ejection fraction (LAEF), is also associated with HF mortality (47) and B-type natriuretic peptide (BNP) levels (50).

The introduction of strain imaging provided further understanding of the role of the LA. Kurt and colleagues (51) found significantly lower levels of LA systolic strain in patients with HFpEF than in patients with LVDD without HF. LA reservoir strain <23% was associated with worse New York Heart Association (NYHA) functional class and elevated estimated pulmonary capillary wedge pressure (PCWP), even in the absence of LA enlargement (52). Reservoir function correlates well with symptoms (53) and peak oxygen consumption at cardiopulmonary exercise testing, even after adjustment for LV and RV longitudinal strain (54). Indeed, a reduction in the reservoir function was found in patients with hypertension (55) and diabetes mellitus (56, 57), which are well-known conditions associated with diastolic dysfunction and HFpEF. A low PALS value is associated with higher disease burden in terms of a history of AF and prior HF hospitalizations (58). However, the interactions between the LV and LA are complex. Some authors question the ability of LA reservoir strain to predict the recurrence of hospitalization for HF after adjustment for LV GLS and E/e' (59). Few studies have explored HF with mid-range ejection fraction (HFmEF). This condition presents higher BNP levels than HFpEF, and worse LA reservoir, conduit, and pump function without a significant difference in the LA size and LV diastolic function (60). The study of LA function should lead to improved therapy management and should not be limited only to the diagnosis and prognosis stratification in patients with suspected or confirmed HFpEF. A better understanding of the reactivity of LA strain parameters to drug treatment may be an essential future endpoint for determining therapeutic efficacy.

### HFrEF

In recent decades, the role of the LA in HFrEF patients has been ignored, while much effort has been dedicated to understanding impaired ventricular function and remodeling. The impact of an enlarged LAV on prognosis has been well-characterized (7, 61), with a prediction of increased mortality and the need for heart transplantation in more advanced phases of HF (62–64). In trials on left ventricular dysfunction (SOLVD) (61), with 1172 HFrEF patients enrolled, the LA dimension was a significant predictor of mortality and HF hospitalization. In particular, in a meta-analysis of 18 studies involving 1157 HFrEF patients, the atrial area was identified as a powerful predictor of death or hospitalization for HF, independent of age, NYHA functional class, LVEF, and restrictive filling pattern (65). Moreover, the maximum LAV was independently associated with death and transplantation in patients with dilated cardiomyopathy (66). Diastolic alteration quantified by E/e' was also associated with long-term mortality in patients with HFrEF (62). However, LV fibrosis and restricted mitral annular motion make this method unreliable for quantifying LV diastolic pressure (67). Tissue-Doppler velocity during atrial contraction provides information on atrial contractile function. When related to LAVi, it gives origin to the left atrial volumetric/mechanical coupling index, a useful predictor of death in HFrEF patients, and functional MR (68). The introduction of strain imaging has enabled a deeper understanding of atrial function. Some authors have proposed PALS as a better predictor of cardiovascular events than LAEF and LA function index (69). The reservoir strain is reduced in HFrEF and HFpEF patients, but HFrEF patients showed a more significant reduction in LA strain proportional to LV GLS (70). Moreover, lower PALS values were associated with adverse events and HF symptoms, and the outcomes remained significant after adjusting for BNP levels, LAVi, E/e' ratio, and LV GLS. Specifically, PALS value <12.9% was correlated with an augmented risk of 30% per year adverse event rate, which decreased to only 4.9% when PALS was above 18.6% per year (71). Cameli et al. (72) demonstrated, in 36 patients with HFrEF who underwent right heart catheterization, that LA systolic strain is best correlated to PCWP and provided the highest diagnostic accuracy in predicting elevated LV end-diastolic pressure. Interestingly, the lack of reserve in LA contractility in patients with HFrEF was associated with right ventricular-to-pulmonary uncoupling during exercise and recovery along with ventilation inefficiency (73). Furthermore, PALS is strongly correlated with functional capacity during exertion and is more depressed in the idiopathic form of dilated cardiomyopathy than in the ischemic one (74).

### Acute HF

In acute HF, PALS is a strong predictor of prognosis regardless of HF phenotype, sex, age, ventricular function, or LAVi (75). Similar to a non-acute setting, PALS is associated with GLS at baseline but decreases disproportionately with congestion; decongestant therapy is correlated with a prompt reduction in LA pressure and immediate improvement in the function of the reservoir, independent of changes in LV GLS, LAV, or MR severity (76). The marked improvement in PALS is independently related to a reduced risk or all-cause or HF readmissions (76). Furthermore, the booster pump function recovers after 6 weeks. This demonstrates that increased atrial afterload is not the only factor that induces atrial dysfunction as concomitant contractile impairment occurs.

#### Atrial Fibrillation

HF increases the risk of developing AF by 10–50% in several ways: dysregulation of intracellular calcium, interstitial fibrosis, and autonomic and endocrine dysfunction (77). Moreover, most HF patients (regardless of LVEF) have enlarged LA and mechanical dysfunction, which secondarily contribute to pulmonary hypertension (PH) and eventually AF development (49). Furthermore, worsening of diastolic function is correlated with a cumulative risk of developing AF, suggesting that the stretch imposed on the atrial cells by increased LV filling pressure may be proarrhythmic. MR is also an important predisposing factor for the onset of arrhythmia due to the pronounced dilatation of the atrial chamber (78, 79). Thus, once established, AF may either contribute to degenerative MR disease progression or unfavorably influence prognosis on its own, or both (80). Among the predisposing factors for the development of AF, advanced age and LA dilation play a predominant role. When patients with degenerative MR receive a surgical indication, the degree of enlargement of the atrial chamber must always be evaluated (81). Therefore, it is crucial to evaluate echocardiographic parameters to identify structural and functional alterations that could predict the onset of AF (82). A large study suggested that echocardiographic parameters associated with a diastolic function such as transmitral peak E wave velocity and A wave VTI and the dimension of LA may predict AF (83). A 43% increase in the risk of developing AF is associated with a 30% increase in maximum LAV, independent of clinical risk factors (84), LVEF, and the severity of diastolic dysfunction (26). Persistent AF forms are associated with higher fibrosis measured by CMR and more depressed LA function, as assessed by strain analysis (85). AF is also associated with an augmented risk of cardiovascular outcomes in HF regardless of EF baseline. The recent onset of AF in HFpEF patients is an adverse prognostic indicator that elevates the risk of adverse events, approaching that of HFrEF patients in sinus rhythm (86).

#### Aortic Stenosis and Mitral Regurgitation

The assessment of LA function in patients with aortic stenosis (AS) is of increasing interest. All three LA phasic functions in AS are significantly lower in patients with severe symptomatic forms of valvular disease than in asymptomatic ones (87). LA booster pump function is strictly correlated to the severity of AS and LVDD (88). Marques-Alves and colleagues (89) demonstrated that LA reservoir strain was closely associated with the aortic valve area and mean transvalvular aortic gradient, whereas, LV GLS was not. This may indicate poor LA compliance, even before LV dysfunction occurs. LA reservoir function is a recognized marker of poor prognosis in patients with AS (90). PALS values of ≤ 21% are associated with a significant risk of cardiac hospitalization, worsening HF, and cardiac death (91). Calin et al. (92) found that LA dysfunction and dilatation were significantly related to PH in patients with severe AS and preserved ventricular systolic performance. In particular, LA booster pump function correlated independently with PH in multivariable analysis. LA systolic strain, as an indicator of LA reverse remodeling, predicts postoperative development of AF (93). It also improves after aortic valve replacement (88), along with reduced LA size. Interestingly, the most remarkable changes have been reported during the first 40 days following intervention (94), with a residual postoperative aortic mean gradient significantly affecting recovery of atrial function (95).

LA dilatation resulting from volume overload is common in chronic MR. It reflects the duration of regurgitation and the severity of valvular disease (96) and has long been considered an essential predictor of adverse cardiovascular outcomes (97, 98). The characterization of atrial function through 2DSTE can offer insights into atrial adaptation to chronic MR. Cameli et al. (99) demonstrated that in a heterogeneous group of patients with asymptomatic mitral valvular prolapse (MVP), global PALS was raised in mild MR due to an increase in LA compliance but decreased linearly with increasing severity. In contrast, these decreases in patients with moderate and severe regurgitation may reflect ultrastructural abnormalities and adverse remodeling. Evaluation of LA function compared to LA size may provide further information on the optimal surgery timing and predict postoperative outcomes. In 87 subjects with degenerative MR enrolled in the randomized EVEREST II trial (100), LA strain modification was correlated with baseline LV and LA function. Changes in LA strain after reduction in regurgitation may reflect a decrease in LA enlargement but may also be affected by the degree of pre-existing LA dysfunction (101). Although, LA volume and function are strongly correlated, modification of LA function appears earlier than cavity remodeling and can be present even if LAV is normal (57). This dissociation between atrial volume and pressure may be explained in the early phases of acute MR by the lack of extracellular LA fibrosis. In this phase, the LA is still compliant and can compensate for atrial volume overload. When compliance is lost, there is an increase in the pulmonary pressure, reflecting the complex underlying interaction of right ventricle–pulmonary circulation uncoupling.

## Left Atrial Strain as a Therapeutic Target

The LA can undergo inverse remodeling after reducing LA pressure and/or LAV overload (49), leading to subsequent improvements in ventricular compliance and function due to LA–LV interdependency (84). Reverse remodeling is possible after treatment for different diseases, such as MR, AF, and hypertension, or following cardiac resynchronization therapy (CRT) (26, 102, 103). In HF patients, administration of angiotensin receptor inhibitors reduces atrial fibrosis, electrical remodeling, and the occurrence of the first episode of AF (104). The secretion of atrial natriuretic peptide (ANP) is also impaired in HF and is correlated with fibrotic alterations in the LA, leading to sodium and fluid retention. However, restoration of endocrine function occurs upon administration of sacubitril/valsartan. In patients with HFrEF, this treatment may promote LA reverse remodeling within 9 months, improving reservoir function and LAEF (105). The degree of recovery is correlated with improvements in atrial mechanical function and reduced LAV. Interestingly, a rapid and significant rise in ANP levels is associated with greater gains in LVEF after therapy, suggesting that the LA may be an indicator of responsiveness to HF therapy (106). Reverse remodeling has been observed even in patients with HFpEF, as demonstrated by a reduction in LAV and BNP levels during therapy with carvedilol, angiotensin receptor, and neprilysin inhibitor (3, 107). Although, CRT improves outcomes and overall survival and is recommended in patients with HFrEF, its efficacy is limited by a high percentage of non-responders. Those patients with maximum LAV > 59.4 ml/m^2^ continue to have increased mortality despite CRT (108). However, patients with lower LAV and mild MR show a more significant response (109).

## Conclusion

As shown in the literature, the LA plays a crucial role in HF. Global LA failure is associated with an increased risk of incident AF, poor exercise tolerance, and increased morbidity and mortality. 2DSTE is a promising technique that allows for the quantification of myocardial deformation and provides additive information about cardiac function. It can detect subtle myocardial damage and has excellent clinical diagnostic and prognostic value for HF evaluation, heart valve disease, and AF. Furthermore, due to its evident accuracy in predicting results and the recent standardization of myocardial deformation imaging, it could be considered as part of risk stratification protocols. Therefore, the assessment of the LA chamber becomes of crucial importance for tailored therapies in different clinical scenarios.

## Author Contributions

MC: conceptualization, review of the literature, writing and review, and editing of the manuscript. DF, SM, and MB: review of the literature and writing. GB and AR: critical revision to scientific content. FR and FG: conceptual guidance, critical revision, and editing of the manuscript. All authors read, commented, and approved the final version of the manuscript.

## Conflict of Interest

The authors declare that the research was conducted in the absence of any commercial or financial relationships that could be construed as a potential conflict of interest.

## Publisher's Note

All claims expressed in this article are solely those of the authors and do not necessarily represent those of their affiliated organizations, or those of the publisher, the editors and the reviewers. Any product that may be evaluated in this article, or claim that may be made by its manufacturer, is not guaranteed or endorsed by the publisher.

## Abbreviations

AF, atrial fibrillationcANP: atrial natriuretic peptide
AS: aortic stenosis
ASE: American Society of Echocardiography
BNP: B-type natriuretic peptide; CCT-cardiac computed tomography
CMR: cardiac magnetic resonance
CRT: cardiac resynchronization therapy
EACVI: European Association of Cardiovascular Imaging
EF: ejection fraction
GLS: global longitudinal strain
HF: heart failure
HFmEF: heart failure with mid-range ejection fraction
HFpEF: heart failure with preserved ejection fraction
HFrEF: heart failure with reduced ejection fraction
LA: left atrium
LAEF: left atrial ejection fraction
LAV: left atrial volume
LAVi: left atrial volume indexed to body surface area
LV: left ventricle/ventricular
LVDD: left ventricle diastolic dysfunction
LVEF: left ventricular ejection fraction
MR: mitral regurgitation
MVP: mitral valve prolapse
NYHA: New York Heart Association
PACS: peak of atrial contraction strain
PALS: peak of atrial longitudinal strain
PCWP: pulmonary capillary wedge pressure
PH: pulmonary hypertension
TDI: tissue Doppler imaging
2DSTE: 2D speckle-tracking echocardiography
2DE: two dimensional echocardiography
3DE: three dimensional echocardiography.
